# Supplementary material for: A 12-Week, Single-Centre, Randomised, Double-Blind, Placebo-Controlled, Parallel-Design Clinical Trial for the Evaluation of the Efficacy and Safety of Lactiplantibacillus plantarum SKO-001 in Reducing Body Fat
Source: Nutrients. 2024 Apr 11;16(8):1137. doi: 10.3390/nu16081137 (PMC11053414; doi:10.3390/nu16081137)
Supplement: Supplementary file 1 [file nutrients-16-01137-s001.zip › nutrients-2944146-supplementary.pdf]

Supplementary Materials

Title: A 12-Week, Single-Centre, Randomised, Double-Blind, Placebo-Controlled, Parallel Design Clinical Trial for the Evaluation of the Efficacy and Safety of *Lactiplantibacillus plantarum* SKO-001 on Reducing Body Fat

Seon Mi Shin, Jeong Su Park, Sang Back Kim, Young Hee Cho, Hee Seo, Hak Sung Lee

Table S1. Raw materials and mixing ratio of the study intervention

Interventional formulation	Raw material	Mixing ratio (%)	Content (mg)
SKO-001	*Lactiplantibacillus plantarum* SKO-001*	80.0	360.00
	Maltodextrin	17.0	76.50
	Magnesium stearate	1.00	4.50
	Silicon dioxide	2.00	9.00
	Total	100.00	450.00
Placebo	Gardenia Yellow	0.4	1.8
	Maltodextrin	96.60	436.70
	Magnesium stearate	1.00	4.50
	Silicon dioxide	2.00	9.00
	Total	100.00	450.00

* *Lactiplantibacillus plantarum SKO-001* contains 2 × 10^{10} CFU of *L. plantarum* SKO-001 per 1 g of corn starch.

Table S2. Endpoint results at 12 weeks from baseline according to the groups.

		**SKO-001 group (N = 45)**	**Placebo group (N = 47)**
Body fat percentage (%)	V2	38.98 ± 5.19	39.49 ± 5.01
	V5	38.79 ± 5.18	39.97 ± 5.19
	V5-V2	−0.19 ± 1.36	0.48 ± 1.23
	p-value	0.357	**0.011***
	p-value	#**0.016***	
Body fat mass, g	V2	26,631.58 ± 4,216.42	27,102.13 ± 3,632.25
	V5	26,462.07 ± 4,242.20	27,452.51 ± 3,924.90
	V5-V2	−169.51 ± 1,168.26	350.38 ± 1,216.37
	p-value	^0.336[1]	**†0.022***
	p-value	**$0.020***	
Fat-free mass, g	V2	44,277.53 ± 7,527.85	44,279.30 ± 7763.1
	V5	44,320.60 ± 7,449.52	44,018.60 ± 8,184.36
	V5-V2	43.07 ± 1,197.40	−260.70 ± 1,233.18
	p-value	†0.631	^0.154
	p-value	0.175	
Body fat percentage (%) (Trunk)	V2	42.46 ± 5.61	42.90 ± 4.83
	V5	42.23 ± 5.51	43.73 ± 4.99
	V5-V2	−0.23 ± 1.80	0.83 ± 1.78
	p-value	†0.388	^0.002***
	p-value	**0.005*****	
Body fat percentage (%) (Android)	V2	44.98 ± 6.51	45.68 ± 5.26
	V5	44.66 ± 6.77	46.59 ± 5.30
	V5-V2	−0.33 ± 2.35	0.91 ± 2.14

	p-value	†0.356	^0.005**
	p-value	0.010***	
Body fat mass, g (Trunk)	V2	15,115.67 ± 2,860.99	15,232.47 ± 2,197.87
	V5	14,997.44 ± 2,814.50	15,533.26 ± 2,329.50
	V5-V2	−118.22 ± 805.98	300.79 ± 851.86
	p-value	†0.331	0.019*
	p-value	0.017*	

SKO-001: *Lactiplantibacillus plantarum* SKO-001-based interventional formulation; V, visit; *$p < 0.05$, **$p < 0.01$, ***$p < 0.001$. #: Independent *t*-test $: Wilcoxon rank sum test, ^: Paired *t*-test, †: Wilcoxon signed-rank test.

Table S3. Endpoint results at 12 weeks from baseline according to the groups .

Parameter	Treatment	Baseline	4 weeks	8 weeks	12 weeks	*p*-value	*p*-value
Weight (kg)	SKO-001	71.27 ± 8.55	71.43 ± 8.58	71.28 ± 8.48	71.13 ± 8.53	0.535	0.726
	Placebo	71.72 ± 8.02	72.09 ± 8.26	72.06 ± 8.55	71.70 ± 8.81	0.949	
	p-value	0.796	0.708	0.665	0.758		
BMI (kg/m^2)	SKO-001	27.27 ± 1.48	27.34 ± 1.59	27.29 ± 1.52	27.24 ± 1.63	0.64	0.888
	Placebo	27.50 ± 1.41	27.64 ± 1.49	27.61 ± 1.61	27.48 ± 1.73	0.835	
	p-value	0.467	0.356	0.331	0.494		
Waist circumference (cm)	SKO-001	92.09 ± 5.49	92.10 ± 5.38	91.82 ± 5.28	91.79 ± 5.50	0.582	0.809
	Placebo	92.65 ± 5.07	92.85 ± 4.52	93.00 ± 4.95	92.67 ± 4.88	0.969	
	p-value	0.69	0.476	0.271	0.421		
Hip circumference (cm)	SKO-001	101.85 ± 4.19	101.43 ± 4.18	101.50 ± 4.24	101.27 ± 4.20	0.064	0.836
	Placebo	102.58 ± 3.75	102.57 ± 3.64	102.47 ± 3.64	102.17 ± 3.70	0.08	
	p-value	0.382	0.169	0.21	0.282		
WHR	SKO-001	0.90 ± 0.05	0.91 ± 0.05	0.90 ± 0.05	0.90 ± 0.05	0.299	0.32
	Placebo	0.91 ± 0.05	0.91 ± 0.05	0.91 ± 0.04	0.91 ± 0.05	0.79	
	p-value	0.845	0.979	0.122	0.289		

SKO-001: *Lactiplantibacillus plantarum* SKO-001-based interventional formulation; BMI, body mass index; WHR, waist/hip ratio.

Table S4. Calorie intake

**Parameter**	**Treatment**	**Baseline**	**12 weeks**	***p*-value**	***p*-value**
Calorie intake (Kcal)	SKO-001	1,719.80 ± 562.47	1565.48 ± 615.87	†0.121	0.754
	Placebo	1,617.66 ± 489.86	1476.92 ± 456.85	^0.104	
	p-value	0.278	0.740		

SKO-001: Lactiplantibacillus plantarum SKO-001-based interventional formulation. $: Wilcoxon's rank sum test ^: Paired t-test †: Wilcoxon's signed-rank test

Table S5. Analysis of participants with calorie intake exceeding 1500 kcal (as of visit 2, baseline)

**Parameters**	Treatment	Baseline	12 weeks	*p*-value	*p*-value
Body fat percentage (%)	SKO-001	39.20 ± 4.91	38.95 ± 5.11	^0.385	#**0.030***
	Placebo	38.40 ± 5.43	38.97 ± 5.66	^0.**021***	
	p-value	$0.710	$0.883		
Body fat mass (g)	SKO-001	27,837.63 ± 4,242.20	27,530.74 ± 4,302.76	†0.645	**$0.004****
	Placebo	27,095.44 ± 3,736.10	27,616.70 ± 4,007.39	**^0.025***	
	p-value	#0.498	#0.940		
LDL-C (mg/dL)	SKO-001	137.48 ± 28.33	127.78 ± 30.23	**0.016***	#**0.012***
	Placebo	121.33 ± 33.93	126.33 ± 36.63	0.241	
	p-value	$0.059	#0.875		
Adiponectin (ng/mL)	SKO-001	10,040.28 ± 6240.01	10,966.44 ± 7,390.67	†0.111	**$0.004****
	Placebo	9,908.54 ± 3641.65	9,083.96 ± 3,717.60	**^0.044***	
	p-value	$0.400	$0.918		

SKO-001: *Lactiplantibacillus plantarum* SKO-001-based interventional formulation; LDL-C, low-density lipoprotein-cholesterol

**p* < 0.05, ***p* < 0.01

#: Independent-samples *t*-test

$: Wilcoxon rank sum test

^: Paired *t*-test

†: Wilcoxon signed-rank test

Number of participants in each group:: SKO 27, Placebo 27

Table S6. Stratification analysis of participants with calorie intake exceeding 1500 kcal (based on average of visits 2 and 5)

**Parameters**	**Treatment**	**Baseline**	**12 weeks**	***p*-value**	***p*-value**
Body fat percentage (%)	SKO-001	38.22 ± 5.73	37.88 ± 5.64	0.227	#**0.017***
	Placebo	38.55 ± 5.74	39.13 ± 5.98	**0.030***	
	p-value	$0.624	$0.289		
Body fat mass (g)	SKO-001	27,056.36 ± 4,191.11	26,581.48 ± 3,995.47	0.078	#**0.007****
	Placebo	27,325.04 ± 3,854.69	27,856.58 ± 4,156.20	**0.039***	
	p-value	#0.816	#0.280		
Body weight (kg)	SKO-001	74.03 ± 8.89	73.42 ± 8.94	**0.034***	**0.023***
	Placebo	74.17 ± 7.84	74.53 ± 8.48	0.266	
	p-value	0.951	0.658		
Body mass index (kg/m²)	SKO-001	27.48 ± 1.45	27.26 ± 1.54	†0.122	**$0.035***
	Placebo	27.58 ± 1.33	27.69 ± 1.50	^0.353	
	p-value	#0.803	#0.328		
LDL-C (mg/dL)	SKO-001	135.84 ± 30.19	128.64 ± 30.98	0.074	#**0.038***
	Placebo	123.75 ± 30.42	129.25 ± 32.76	0.237	
	p-value	$0.158	#0.947		
Adiponectin (ng/mL)	SKO-001	10,472.46 ± 5939.50	11,229.60 ± 6,580.48	0.092	#**0.005****
	Placebo	9,545.21 ± 3544.99	8,713.62 ± 3,399.07	**0.014***	
	p-value	$0.992	4.399		

SKO-001: *Lactiplantibacillus plantarum* SKO-001-based interventional formulation; LDL-C, low-density lipoprotein-cholesterol

*p < 0.05, **p < 0.01

#: Independent-samples *t*-test

$: Wilcoxon rank sum test

^: Paired *t*-test

†: Wilcoxon signed-rank test

Number of participants in each group: SKO 25, Placebo 24

Table S7. Stratification analysis of participants with physical activity of <3000 METs/w at visit 2 (baseline)

**Parameters**	**Treatment**	**Baseline**	**12 weeks**	***p*-value**	***p*-value**
Body fat percentage (%)	SKO-001	38.85 ± 5.25	38.67 ± 5.30	^0.450	#**0.027***
	Placebo	40.18 ± 4.45	40.72 ± 4.58	^0.**017***	
	p-value	$0.295	$0.**106**		
Body fat mass (g)	SKO-001	26,575.41 ± 4,506.95	26,352.65 ± 4,500.42	^0.271	**$0.019***
	Placebo	27,669.45 ± 3,746.11	28,061.85 ± 4,049.47	†0.050	
	p-value	#0.272	#0.099		
TG (mg/dL)	SKO-001	112.49 ± 39.77	126.24 ± 64.56	^0.092	**$0.043***
	Placebo	119.15 ± 60.40	104.70 ± 36.02	†0.097	
	p-value	$0.995	$0.311		
Adiponectin (ng/mL)	SKO-001	10,315.11 ± 5,407.45	10,705.14 ± 6,133.35	†0.808	**$0.048***
	Placebo	10,776.39 ± 5,476.20	10,123.74 ± 5,656.39	^0.102	
	p-value	$0.657	$0.726		

SKO-001: *Lactiplantibacillus plantarum* SKO-001-based interventional formulation; TG, triglycerides.
#: Independent-samples *t*-test
$: Wilcoxon rank sum test
^: Paired *t*-test
†: Wilcoxon signed-rank test
Number of participants in each group: SKO 37, Placebo 33

Table S8. Stratification analysis of participants with physical activity of <3,000 METs/w (based on the average of visits 2 and 5)

Components	Treatment	Baseline	12 weeks	*p*-value	*p*-value
Body fat percentage (%)	SKO-001	39.12 ± 5.38	38.94 ± 5.43	^0.477	#**0.026***
	Placebo	40.67 ± 3.92	41.23 ± 4.10	^0.**018***	
	p-value	$0.199	$0.**065**		
Body fat mass (g)	SKO-001	26,726.35 ± 4,437.41	26,554.11 ± 4,409.98	^0.398	**$0.039***
	Placebo	27,527.77 ± 3,596.41	27,867.74 ± 3,976.36	†0.090	
	p-value	#0.402	#0.188		
TG (mg/dL)	SKO-001	116.32 ± 38.32	128.22 ± 63.86	^0.261	**$0.016***
	Placebo	122.00 ± 59.55	102.74 ± 36.65	†0.005*	
	p-value	$0.933	$0.121		

SKO-001: *Lactiplantibacillus plantarum* SKO-001-based interventional formulation; TG, triglycerides.

*$p < 0.05$

#: Independent-samples *t*-test

$: Wilcoxon rank sum test

^: Paired *t*-test

†: Wilcoxon signed-rank test

Number of each group subject: SKO 37, Placebo 35

Table S9. Adverse events (safety set)

	SKO (n = 50)	Placebo (n = 50)	Total
Total adverse event cases	46	46	92
At least one adverse event, n (%)	27 (54.0)	28 (56.0)	55 (55.0)
Serious adverse event, n (%)	0 (0.0)	1 (2.0)	1 (1.0)
Type of serious adverse event			
- death	0	0	0
- hospitalisation	0	1	1
- extended hospitalisation	0	0	0
- life-threatening event	0	0	0

SKO: *Lactiplantibacillus plantarum* SKO-001-based interventional formulation

Table S10. Haematology (safety set)

Parameter	Treatment	Baseline	12 weeks	*p*-value	*p*-value
WBC (×10³/μL)	SKO-001	6.15 ± 1.82	5.78 ± 1.52	0.040*	#0.124
	Placebo	5.93 ± 1.71	5.87 ± 1.39	0.910	
	p-value	$.581	$.516		
RBC (×10⁶/μL)	SKO-001	4.90 ± 0.35	4.80 ± 0.35	0.000***	#0.461
	Placebo	4.87 ± 0.38	4.81 ± 0.39	0.081	
	p-value	$.735	$.729		
Haemoglobin (g/dL)	SKO-001	14.75 ± 1.02	14.54 ± 1.10	^0.006**	$0.865
	Placebo	14.12 ± 1.51	13.99 ± 1.58	†0.048*	
	p-value	#.016*	$.290		
Haematocrit (%)	SKO-001	44.11 ± 2.88	43.35 ± 2.88	0.001	#0.862
	Placebo	42.84 ± 3.66	42.12 ± 3.86	0.027	
	p-value	#.056	$.263		
Platelet (×10³/μL)	SKO-001	289.72 ± 56.38	283.06 ± 55.71	0.089	#0.313
	Placebo	314.00 ± 67.45	312.00 ± 70.76	0.956	
	p-value	#.054	#.028*		
Neutrophil (%)	SKO-001	53.82 ± 7.93	52.71 ± 8.21	^0.248	$0.028*
	Placebo	55.01 ± 8.11	56.88 ± 9.31	†0.070	
	p-value	#.462	#.021*		
Lymphocyte (%)	SKO-001	35.95 ± 7.66	37.07 ± 8.00	^0.186	$0.027*
	Placebo	35.20 ± 7.13	33.79 ± 8.17	†0.082	
	p-value	#.612	#.049*		
Monocyte (%)	SKO-001	7.22 ± 1.75	7.45 ± 1.86	†0.691	$0.449
	Placebo	6.88 ± 1.63	6.73 ± 1.58	^0.377	
	p-value	#.321	$.091		
Eosinophil (%)	SKO-001	2.38 ± 1.51	2.17 ± 1.48	0.140	$0.986
	Placebo	2.27 ± 1.58	2.06 ± 1.53	0.123	

	p-value	$.764	$.576		
Basophil (%)	SKO-001	0.61 ± 0.25	0.60 ± 0.26	0.870	$0.126
	Placebo	0.64 ± 0.42	0.54 ± 0.26	0.058	
	p-value	$.414	#.267		

SKO-001: *Lactiplantibacillus plantarum* SKO-001-based interventional formulation; WBC: White blood cell; RBC: Red Blood cell

*$p < 0.05$, **$p < 0.01$, $p < 0.001$

#: Independent-samples t-test, $: Wilcoxon rank sum test, ^: Paired t-test,†: Wilcoxon signed-rank test

Number of each group participants: SKO 50, Placebo 50

Table S11. Blood biochemistry (safety set)

Parameter	Treatment	Baseline	12 weeks	*p*-value	*p*-value
AST (U/L)	SKO-001	25.44 ± 8.28	22.92 ± 6.67	^0.006**	$0.548
	Placebo	25.12 ± 5.43	24.10 ± 10.70	†0.018*	
	p-value	$.604	$.845		
ALT (U/L)	SKO-001	23.26 ± 11.24	20.71 ± 9.26	0.013*	$0.401
	Placebo	22.26 ± 9.27	21.45 ± 11.64	0.135	
	p-value	$.975	$.851		
γ-GT (U/L)	SKO-001	32.20 ± 49.39	28.65 ± 32.05	0.046*	$0.184
	Placebo	23.40 ± 13.42	22.37 ± 12.85	0.739	
	p-value	$.593	$.828		
ALP (U/L)	SKO-001	68.28 ± 22.77	64.50 ± 17.24	0.001	#0.302
	Placebo	63.00 ± 14.62	59.94 ± 14.92	0.027	
	p-value	$.322	$.208		
Total Bilirubin (mg/dL)	SKO-001	0.74 ± 0.24	0.80 ± 0.27	0.064	#0.684
	Placebo	0.67 ± 0.27	0.75 ± 0.23	0.012*	
	p-value	$.067	$.457		
BUN (mg/dL)	SKO-001	14.33 ± 3.95	14.06 ± 3.56	^0.816	$0.860
	Placebo	13.68 ± 3.54	13.45 ± 3.08	†0.889	
	p-value	#.386	#.364		
Creatinine (mg/dL)	SKO-001	0.79 ± 0.15	0.79 ± 0.15	0.550	#0.121
	Placebo	0.77 ± 0.15	0.79 ± 0.14	0.129	
	p-value	$.258	$.888		
Glucose (mg/dL)	SKO-001	89.20 ± 10.99	92.02 ± 10.55	0.147	#0.648
	Placebo	88.96 ± 10.53	90.22 ± 10.20	0.355	
	p-value	#.911	#.396		
hs-CRP (mg/dL)	SKO-001	1.36 ± 2.15	0.89 ± 0.80	0.479	$0.754
	Placebo	1.79 ± 2.47	1.34 ± 1.98	0.240	

	p-value	$.537	$.885		
C-peptide (ng/mL)	SKO-001	2.13 ± 1.06	1.95 ± 0.93	0.060	$0.525
	Placebo	1.87 ± 0.50	1.81 ± 1.47	0.006**	
	p-value	$.676	$.219		

SKO-001: *Lactiplantibacillus plantarum* SKO-001-based interventional formulation; AST: Aspartate aminotransferase; ALT: Alanine aminotransferase; γ-GT: Gamma-glutamyltransferase; ALP: Alkaline phosphatase; BUN: Blood urea nitrogen; hs_CRP: High-sensitivity C-reactive protein

*$p < 0.05$, **$p < 0.01$

#: Independent-samples t-test, $: Wilcoxon rank sum test,^: Paired t-test ,†: Wilcoxon signed-rank test

SKO-001, n = 50; Placebo, n = 50

Table S12. Urinalysis (safety set)

Parameter	Treatment	Baseline	12 weeks	*p*-value	*p*-value
pH	SKO-001	5.67 ± 0.79	5.66 ± 0.70	†0.913	0.662
	Placebo	5.79 ± 0.89	5.94 ± 0.98	^0.366	
	p-value	.524	.265		
Specific Gravity	SKO-001	1.02 ± 0.01	1.02 ± 0.01	^0.748	0.939
	Placebo	1.02 ± 0.01	1.02 ± 0.01	†0.918	
	p-value	.787	.767		

SKO-001: *Lactiplantibacillus plantarum* SKO-001-based interventional formulation; pH: Hydrogen ion concentration index
SKO-100, n = 50; Placebo, n = 50
: Wilcoxon's rank sum test

^ : Paired t test

† : Wilcoxon's signed rank test

Table S13. urinalysis about Comparing Protein, Glucose, WBC, and RBC in **Normal/Abnormal** categorical format

**Parameter**	**Treatment**	**Baseline (Normal/Abnormal)**	**12 weeks (Normal/Abnormal)**
Protein	SKO-001	45/5	39/9
	Placebo	44/6	42/7
	p-value	‡1.000	‡0.750
Glucose	SKO-001	50/0	48/0
	Placebo	50/0	49/0
	p-value	&1.000	&1.000
WBC	SKO-001	47/3	47/1
	Placebo	48/2	47/2
	p-value	&1.000	&1.000
RBC	SKO-001	28/22	32/16
	Placebo	27/23	31/18
	p-value	‡1.000	‡0.890

SKO-001: *Lactiplantibacillus plantarum* SKO-001-based interventional formulation; WBC: White blood cell; RBC: Red Blood cell

‡: Chi-square test , &: Fisher’s exact test

SKO-100, n = 50; Placebo, n = 50

Table S14. Vital sign (safety set)

Parameter	Treatment	Baseline	12 weeks	*p*-value	*p*-value
BP (systolic; mmHg)	SKO-001	127.76 ± 9.77	127.76 ± 8.97	0.918	#0.958
	Placebo	126.28 ± 9.08	126.41 ± 11.53	0.865	
	p-value	$.366	$.667		
BP (diastolic; mmHg)	SKO-001	77.78 ± 8.63	78.76 ± 7.89	0.312	$0.143
	Placebo	78.36 ± 7.63	77.55 ± 8.26	0.173	
	p-value	$.775	#.462		
Pulse (bpm)	SKO-001	77.90 ± 8.32	75.35 ± 9.00	0.052	#0.615
	Placebo	78.18 ± 7.88	76.43 ± 8.40	0.134	
	p-value	#.863	#.540		
BT (°C)	SKO-001	36.52 ± 0.17	36.51 ± 0.21	0.194	#0.561
	Placebo	36.57 ± 0.24	36.52 ± 0.22	0.719	
	p-value	$.431	$.223		

SKO-001: *Lactiplantibacillus plantarum* SKO-001-based interventional formulation; BP: Blood Pressure; BT: Body Temperature

#: Independent-samples t-test $: Wilcoxon rank sum test ^: Paired t-test †: Wilcoxon signed-rank test

SKO-100, n = 50; Placebo, n = 50

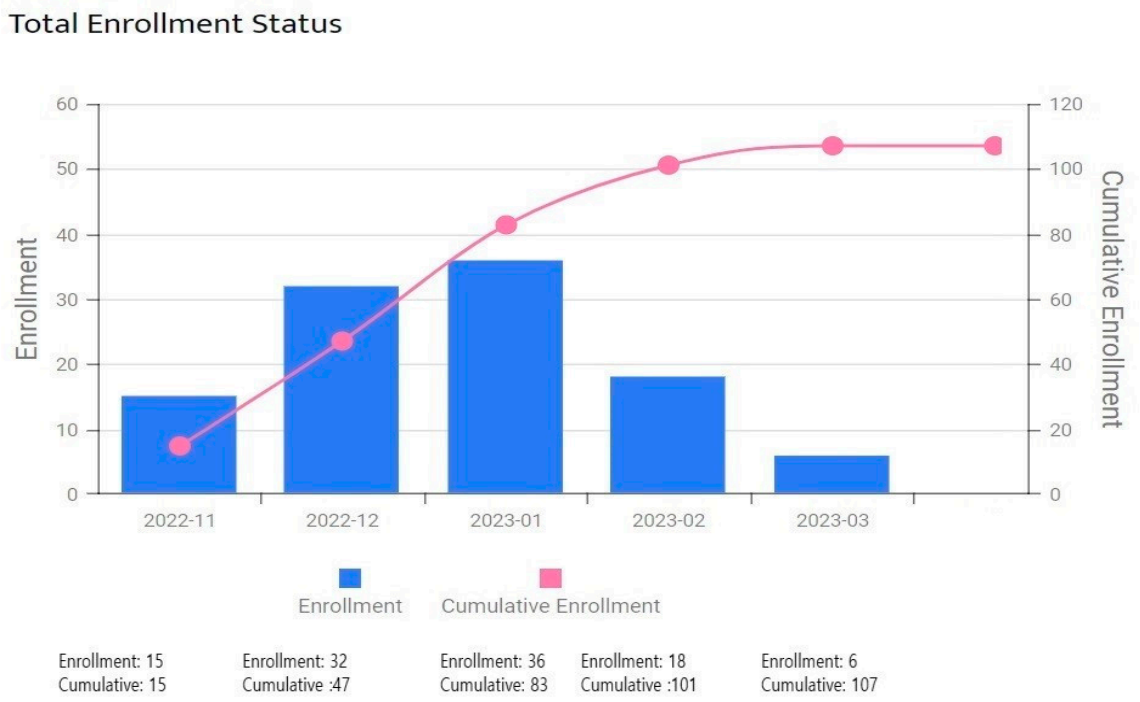

Figure S1. Total enrollment status

Table S15. Seasonal distribution of enrolments

Group	Fall	Winter	Spring	Total
SKO-001	4(8%)	43(86%)	3(6%)	50100%)
placebo	2(4%)	45(90%)	3(6%)	50(100%)

Data are presented as N and %.   Abbreviations : SKO-001: Lactiplantibacillus plantarum SKO-001-based interventional formulation.

Table S16. Abbreviations and glossary of terms

ALP	Alkaline phosphatase
ALT	Alanine aminotransferase
ANCOVA	Analysis of covariance
AST	Aspartate aminotransferase
BP	Blood Pressure
BT	Body Temperature
BUN	Blood urea nitrogen
SKO-001	Lactiplantibacillus plantarum SKO-001
CFU	Colony-forming units
CRP	C-reactive protein
CT	Computed tomography
DEXA	Dual-energy X-ray absorptiometry
GPAQ	Global Physical Activity Questionnaire
γ-GT	Gamma-glutamyltransferase
HDL-C	High-density lipoprotein-cholesterol
HFD	High-fat diet
HFHF	High-fat, high-fructose
Hs-CRP	High-sensitivity C-reactive protein
IPAQ	International Physical Activity Questionnaires
IRB	Institutional review board
LDL-C	Low-density lipoprotein-cholesterol
MET	Metabolic equivalent of task
NAFLD	Non-alcoholic fatty liver disease
pH	Hydrogen ion concentration index
RBC	Red blood cell

SAE	Serious adverse event
SAS	Safety analysis set
TG	Triglyceride
VSR	Visceral fat area/subcutaneous fat area ratio
WBC	White blood cell
WHR	Waist/hip ratio

Advertisements_in_English

Recruiting human subjects for a dietary supplement for body fat reduction

A 12-week, Single-Center, Randomized, Double-Blind, Placebo-Controlled, Parallel-Design Human Application Study to Evaluate the Efficacy and Safety of *Lactobacillus plantarum* SKO-001 in Body Fat Reduction.

■Human application study objectives

This study was designed to evaluate the efficacy and safety of *Lactiplantibacillus plantarum* SKO-001 by observing changes in body fat-related markers when adult male and female subjects consumed the test food (*Lactiplantibacillus plantarum* SKO-001) or control food for 12 weeks. This study aimed to evaluate the efficacy and safety of *Lactiflavacillus plantarum* SKO-001.

■ Human Application Test Methods

Participants will be randomized in a 1:1 ratio to either the treatment or control group.

The duration of participation was 12–14 weeks, with a total of 5–6 visits (additional visits may be scheduled depending on the subject's condition).

Vital signs, physical measurements, blood tests, urinalysis, diet, physical activity, and lifestyle questionnaires were administered at each visit.

■Who can participate

Adult men and women 19 years of age or older and under 65 years of age

Body mass index of more than 25.0 kg/m^2 and less than 30.0 kg/m^2

Voluntarily decide to participate in this human clinical trial and sign an informed consent form

■ Benefits to Participants and Possible Risks and Discomforts

All participants in this study will be paid for transportation (participation fee).

No known side effects were anticipated in either the treatment or control group; however, unexpected side effects may occur.

You may also experience inconveniences from dual-energy X-ray absorptiometry (DEXA), computed tomography (CT), and blood tests.

■How to get involved and call us

If you would like more information about the possible side effects and study methods or would like to apply for this study, please contact the clinical researcher at the Department of Oriental Medicine, Jecheon Oriental Hospital, Seymyeong University at 043-649-1868.

■Human Application Test Institution and Person in Charge

Seymung University Jecheon Korean Medicine Hospital (66, Seymyeong-ro, Jecheon-si, Chungcheongbuk-do) Prof. Shin, Shin-Mi (043-649-1873)

■ Sponsor

Kolmar BnH (61, Hunneung-ro 8-gil, Seocho-gu, Seoul, Korea, 02-3459-5822)
